# Establishing community models as the underpinning of neuroscience: linking computational and experimental data

**DOI:** 10.1186/1471-2202-12-S1-P16

**Published:** 2011-07-18

**Authors:** Hugo Cornelis, Allan D Coop, Armando L Rodriguez, Dave Beeman, James M Bower

**Affiliations:** 1 Department of Neurophysiology, Catholic University of Leuven, Leuven, Belgium; 2 Merindah Energy Ltd, Freemansreach, NSW, 2756, Australia; 3 Research Imaging Institute, University of Texas Health Science Center at San Antonio, TX, 78229, USA; 4 Department of Electrical, Computer, and Energy Engineering, University of Colorado, Boulder, CO 80309, USA

## 

In Physics, as in other “hard sciences”, both research and education are built around systems and their associated models that are accepted as fundamental to the field. This is not the case within Neuroscience, although the existence of simulation systems like GENESIS and NEURON are producing an informal propagation of particular models between laboratories. One of the core tenants of the reconfiguration of GENESIS is that fully developed community models will not exist until the process of building, evaluating, and propagating models is incorporated into the structure of the simulation system itself. To be successful, such systems will also have to provide explicit links to experimental data and procedures.

GENESIS 3.0 (G-3) conforms to the CBI federated software architecture [1]. It is explicitly constructed around an ideal user workflow [2] that provides an organizing principle for simulator use. The modular nature of the system separates low-level numeric data from high-level biological representations, as well as the data and control functions. This facilitates the interface of different databases to significantly enhance two broad categories of user and developer experience. One is organized around an ideal user workflow while the other supports a multi-level documentation system that informs user and developer concerns. We show how this information infrastructure supports scientific communication through the development of community models that can then integrate computational models and experimental neuroscience.

G-3 documentation is organized into seven levels, each served by a single repository: (1-2) Introductory material and user guides, (3-4) Automated use cases and technical guides, (5-7) Algorithm, API and hyperlinked source code documentation. Both within each repository and across the different levels, documents are organized by a user defined tagging system that creates horizontal and vertical reader flows. This simplifies the communication of computational models by separating the technical aspects of a model from its scientific content and interpretation.

The dynamic creation of horizontal and vertical reader flows allows to reinterpret the capabilities of this documentation system from the perspective of a model publication workflow. The lowest levels (Levels 5-7) describe purely technical components of model implementation entirely devoid of scientific interpretation. Higher levels communicate the nontechnical scientific narratives generated by both computational and experimental projects. For example, Level 4-3: Explain biological mechanisms obtained by computational studies, community models, and experimental research, Level 2: Integrates results of computational and experimental neuroscience, and Level 1: General introductions and reviews of neuroscience. This results in a communication gradient where increasing detail about any aspect of a publication is accessible from a given level. We show examples of (1) Horizontal workflows, e.g. model lineage, and (2) Vertical workflows, e.g. “drilling down” from the biological concepts of a model to increasingly technical details of model implementation or, vice-versa, starting from technical implementations going up to their uses in models and simulations and their scientific interpretation. These are the first steps in the implementation of a model-based publication system for scientific communication [3].

Here, we present a new view of the G-3 documentation system as a model sharing system that not only incorporates the models and their interpretation, but also their technical and algorithmic implementation and their relationship with the experimental data on which they are based. Employed in this way, the documentation system also enhances G-3 to support the integration and communication of computational and experimental neuroscience.

This research was supported by NSF grant HRD-0932339 to the University of Texas at San Antonio. Hugo Cornelis is supported by the CREA Financing program (CREA/07/027) of the K.U.Leuven, Belgium. Hugo Cornelis, Armando L. Rodriguez and Allan D. Coop are supported by NIH grant 3 R01 NS049288-06S1 to James M. Bower.
